# Prediction of Cardiovascular Disease Events From the Photoplethysmograph Waveform

**DOI:** 10.1161/JAHA.124.040237

**Published:** 2025-12-03

**Authors:** Ye Li, Harriet Godwin, Marina Cecelja, Kevin O'Gallagher, Ajay Shah, Abdel Douiri, Phil Chowienczyk

**Affiliations:** ^1^ British Heart Foundation Centre School of Cardiovascular and Metabolic Medicine and Sciences, King’s College London London UK; ^2^ School of Population and Life Course Sciences King’s College London UK

**Keywords:** cardiovascular disease events, cardiovascular risks, feature extraction, photoplethysmography, wearables, Cardiovascular Disease, Risk Factors

## Abstract

**Background:**

A photoplethysmographic pulse waveform (PPG) can be obtained optically by digital health technologies such as a smartphone or wearable devices. Features of the PPG may provide prognostic information additive to blood pressure.

**Methods and Results:**

We performed comprehensive feature extraction of PPGs recorded during 2009 to 2010 in 114 884 participants in UK Biobank and examined the association of individual and panel features with incident cardiovascular disease (CVD) events over approximately 10 years of follow‐up. A total of 9242 participants developed CVD events. There were 3378 deaths, with CVD the primary cause of death in 417 participants. In a penalized Cox proportional hazards model, after adjustment for classical risk factors (age, sex, body mass index, ethnicity, smoking, presence of diabetes, total cholesterol/high‐density lipoprotein cholesterol ratio, systolic blood pressure, and medication), PPG indices that were most strongly associated with CVD events included systolic time, the area under the systolic portion of the PPG, and curvature of the mid‐late systolic PPG with standardized hazard ratios of 1.43 (95% CI, 1.28–1.61), 0.79 (95% CI, 0.72–0.88), and 0.80 (95% CI, 0.74–0.86), respectively. The addition of PPG indices to classical risk factors increased the prediction of CVD events and this was more marked in younger compared with older people.

**Conclusions:**

These results suggest that the PPG features related to systolic performance and that may be related to preclinical heart failure, offer important prognostic information. Further studies to identify the physiological properties to which they relate and to optimize their use in risk prediction are merited.

**Nonstandard Abbreviations and Acronyms**
PPG: photoplethysmographic pulse waveform

**Clinical Perspective**

**What Is New**

- Features of the systolic portion of the photoplethysmography waveform that can be derived from smart watches and other wearables predict incident cardiovascular events.

**What Are the Clinical Implications**

- Photoplethysmographic features related to systolic performance that may be related to preclinical heart failure may offer important prognostic information. Further studies to identify the physiological properties to which they relate and to optimize their use in risk prediction are merited.

An optically derived photoplethysmographic pulse waveform (PPG, Figure 1) is the sensing technology used by “wearable” devices such as smart watches to derive physiological data.^1^ The most notable applications of the PPG to date have been to detect atrial fibrillation^2^, ^3^ to measure heart rate variability,^4^ detect circulatory arrest,^5^ and (with calibration from a standard noninvasive blood pressure [BP] monitor) to estimate BP.^6^ Although the amplitude of the PPG depends on local factors determining the perfusion of tissue in the immediate vicinity of the light source and detector, the contour of the pulse is largely determined by the interaction of the left ventricle with the peripheral vasculature and thus captures information on cardiovascular properties.^7^ That physiological features of the PPG might be indicative of aspects of cardiovascular health has been recognized for many years. In the Framingham study visual classification of PPG morphology was associated with incident cardiac events^8^ and the upslope of the initial portion of the PPG has recently been associated with cardiovascular outcomes.^9^ Deep learning techniques have confirmed an association of PPG morphology to incident cardiac events,^10^ but understanding of the link between PPG morphology, cardiovascular physiology and outcomes is limited. Previous measurements derived from characteristic fiducial points of the PPG (Figure 1)^11^, ^12^, ^13^, ^14^, ^15^, ^16^ have been related to vascular stiffening and cardiovascular aging^7^, ^17^ and could provide insight into the prognostic application and impact of the PPG in detecting cardiovascular risk and disease.^18^ Features obtained from the second derivative of the PPG such as the sequential peaks of the second derivative (a, b, c, and d, Figure 1) are also highly correlated with chronological age^17^ and the b and c peaks discriminate between subjects with and without hypertension,^19^ suggesting that they reflect cardiovascular aging. Most PPG features are associated with heart rate and BP^20^ and an unresolved question has been to what extent the association of PPG features with cardiovascular risk are mediated through other cardiovascular risk factors such as heart rate and BP. The objective of the present study was to perform comprehensive “feature extraction” from the PPG recorded in the UK Biobank study and to examine how individual features and combinations of features predict incident cardiovascular events, cardiac death, and all‐cause mortality while adjusting for BP and other conventional risk factors for cardiovascular disease.

**Figure 1:**
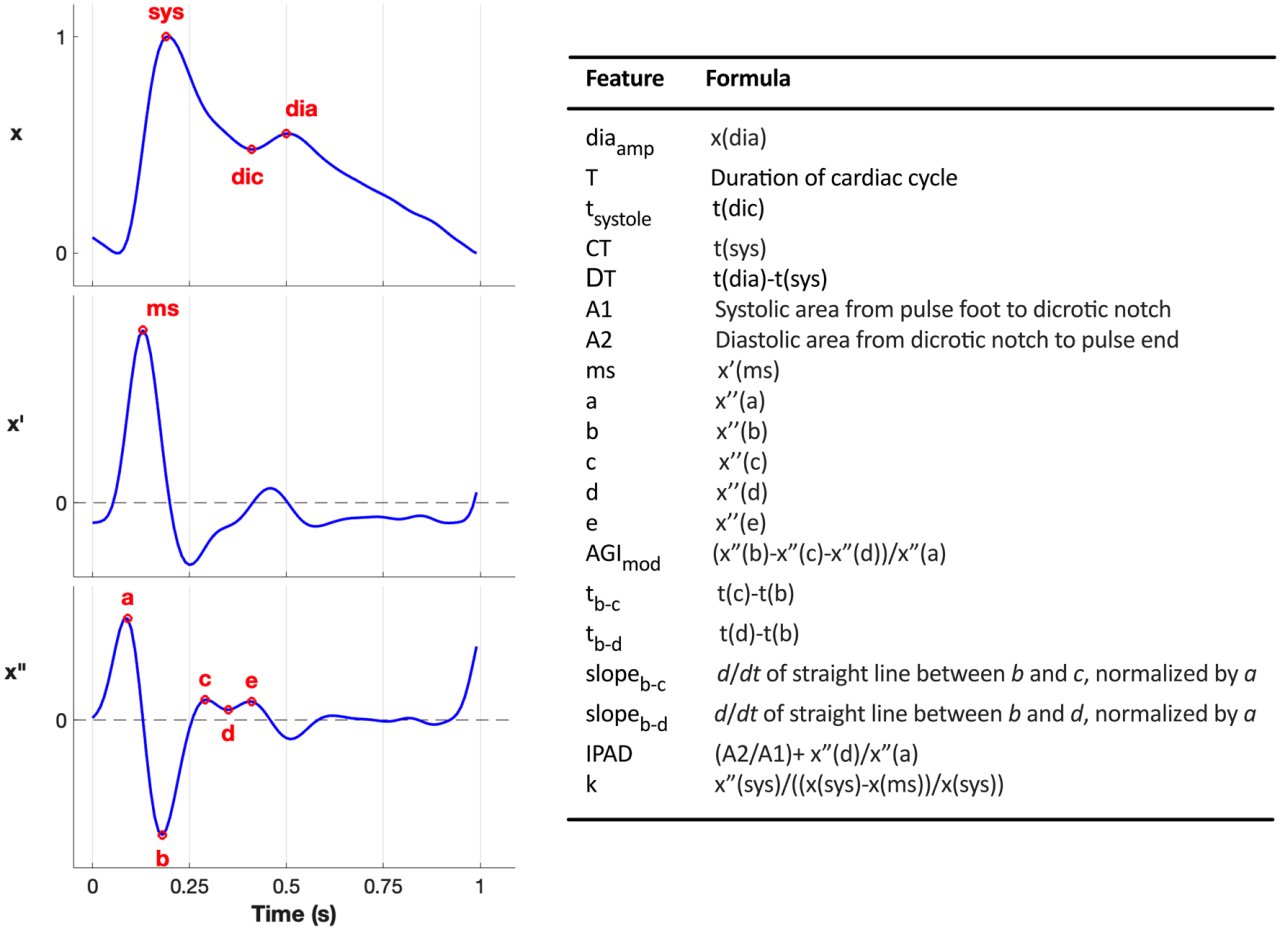
Extracting PPG features by detection of fiducial points on the PPG waveform x, and its first x′ and second x″ derivatives. a indicates amplitude of point a on x″; A1, systolic area from pulse foot to dicrotic notch; A2, diastolic area from dicrotic notch to pulse end; AGI_mod_, modified aging index; b, amplitude of point b on x″; c, amplitude of point c on x″; CT, time of systolic peak; diaamp, amplitude of diastolic peak; d, amplitude of point d on x″; DT, time between diastolic and systolic peaks; e, amplitude of point e on x″; IPAD, ratio of diastolic to systolic area plus ratio of amplitudes of points d to a; k, stiffness constant; ms, amplitude of peak of first derivative of PPG signal (x′); PPG, photoplethysmographic pulse waveform; slope_b‐c_, d/dt of straight line between points b and c on x″, normalized by amplitude of point a; slope_b‐d_, d/dt of straight line between points b and d on x″, normalized by amplitude of point a; T, duration of cardiac cycle; t_systole_, duration of systole; t_b‐c_, time between points b and c on second derivative of PPG signal (x″); and t_b‐d_, time between points b and d on x″.

## METHODS

### UK Biobank Participants and Consent

UK Biobank is a large community‐based prospective study with over half a million UK participants aged from 40 to 69 between 2006 and 2010. Detailed information is available elsewhere. All participants gave written consent and the consent form is available at http://www.ukbiobank.ac.uk/wp‐content/uploads/2011/06/Consent_form.pdf. This research was conducted under UK Biobank Application Number 55014. The raw PPG data and the relevant individual participant data can be requested through UK Biobank (www.ukbiobank.ac.uk).

### Participant Characteristics

Ethnicity, age, sex, height, weight, smoking status, medical history, and medications were recorded during the initial assessment. Brachial systolic BP (SBP) and diastolic BP were measured during the same visit using an automated device (Omron 705, OMRON Healthcare Europe B.V. Kruisweg 577 2132 NA Hoofddorp). A manual sphygmomanometer was used if the standard automated device could not be employed. An appropriate BP cuff was selected based on the measured circumference of the midpoint of the upper arm. Two consecutive measurements were taken sequentially on the left upper arm by a registered nurse with the participant quietly seated and with no restrictive clothing to the left arm. The right arm was used only if the left was not practical (for further details see http://www.biobank.ndph.ox.ac.uk/showcase/showcase/docs/Bloodpressure.pdf). Laboratory measurements including total cholesterol and high‐density lipoprotein (HDL) cholesterol were obtained from venous blood taken at the time of the first visit.

### PPG Measurements

The PPG was recorded using the PulseTrace PCA2 device (CareFusion, San Diego, CA, Biobank UK, Field‐ID 4205), the same PulseTrace PCA2 device being used at all centers. Measurements were obtained with participants seated during the measurement of BP with a fingertip sensor placed on the index finger of the arm not being used for BP measurement. The waveform was allowed to stabilize on a visual display for approximately 1 minute and then averaged over 10 to 15 seconds to provide an ensemble average of the waveform from individual cardiac cycles. Waveforms were normalized with respect to amplitude and the time axis was expressed in seconds. PPG features were extracted using previously validated software.^21^ This software identifies fiducial points in the PPG and its derivatives and provides ~30 indices derived from these points, some of which are combinations of simpler indices or measures obtained from multiple fiducial points. Definitions of the indices that were extracted together with the original reference are provided in Table S1. To reduce colinearity of PPG indices, those that were derived from other indices (eg, as a difference or ratio) were excluded as indicated in Table S1. Also, heart rate was not included because it is inversely related to pulse period, which was used in preference to heart rate because some PPG indices related to time points in the PPG have previously been expressed as a ratio of pulse period.^11^, ^22^ Stiffness index, which has been studied previously,^23^ was not included but the time between the first and second peak of the PPG from which the stiffness index is derived was included. However, for comparison with previous work we did include the PPG aging index derived from several features of the PPG.^14^ In total 20 PPG indices were examined for prediction of cardiovascular events and mortality (Figure 1).

### Ascertainment of Cardiovascular Events and Mortality

Cardiovascular disease (CVD) events: myocardial infarction, diagnosis of coronary heart disease excluding myocardial infarction, heart failure, and stroke were recorded in Hospital Episode Statistics (“Spell and Episode” category), encoded according to the *International Classification of Diseases, Ninth Revision* (*ICD‐9*) and *Tenth Revision* (*ICD‐10*). Cardiovascular death was defined as occurring where cause of death was coded as one of the following according to the *ICD‐10*: myocardial infarction (I21‐23, I252), coronary heart disease (I21‐25, Z951, Z955), heart failure (I110, I130, I132, I50), and stroke (I60, I61, I629, I63, I64, I678, I690, I693, G951, H341, H342, S066). Table 1 shows the definitions used. Date of event is defined as the first date of hospitalization with primary diagnosis. Date of censoring was either 10 years from initial assessment for each participant, time of first event, date lost to follow‐up, or date of death. As described at http://biobank.ndph.ox.ac.uk/showcase/refer.cgi?id=115559, all UK Biobank participants were flagged from the date of their recruitment into the study with the data providers. Date of death, the primary and contributory causes of death (coded using the *ICD‐10* system) were obtained from the National Health Service England for participants in England and Wales and from the National Health Service Central Register for participants in Scotland. Participant follow‐up for mortality started at inclusion in the UK Biobank study and was censored on March 30, 2020.

**Table 1:** Variable Definitions Used in UK Biobank

Variable	*ICD‐9*	*ICD‐10*
Cardiovascular disease	3361, 36231, 36232, 39‐44	I00‐I78, G951, H431, H432, O10, S066, Z951, Z955
Coronary heart disease	410, 412, 414	I21–25, Z951, Z955
Myocardial infarction	410, 412	I21–I23, I252
Heart failure	428	I110, I130, I132, I50
Hypertension	401–405	I10–I13, I15, O10
Stroke	3361, 36231, 36232, 430, 431, 4329, 43301, 43311, 43331, 43381, 43391, 434, 436	I60, I61, I629, I63, I64, I678, I690, I693, G951, H341, H342, S066

*ICD‐9* indicates *International Classification of Diseases, Ninth Revision*; and *ICD‐10*, *International Classification of Diseases, Tenth Revision*.

### Statistical Analysis

Subjects who were alive at the end of the follow‐up period without having experienced a cardiovascular event (March 30, 2020) were treated as right censored. The relationship between individual PPG indices with time to cardiovascular event, cardiovascular mortality, and all‐cause mortality was investigated using Cox proportional hazard models adjusted for classical risk factors including ethnicity, age, sex, body mass index (BMI), smoking status, presence of diabetes, total‐cholesterol to HDL‐cholesterol ratio, mean SBP, and use of antihypertensive medication. The assumption of proportional hazards was checked using graphical diagnostics based on the scaled Schoenfeld residuals for individual PPG indices. Kaplan–Meier survival curves were plotted for outcomes stratified by quartiles of PPG indices that were strongest in predicting outcomes.

A penalized Cox proportional hazards model was used to identify indices contributing to the prediction of outcomes. This was done using an elasticnet proportional hazards model, using the Python package of sksurv.linear_model.CoxnetSurvivalAnalysis. A range of values of elasticnet mixing parameter were fine tuned using 5‐fold cross‐validation by GridSearchCV using the sklearn.model_selection package. Hazard ratios (HRs) and 95% CI for outcomes were reported. The concordance index (C‐statistic, predictive accuracy), which measures the proportion of pairs where the observation with the higher survival time has the higher probability of survival as predicted by the model, was used as the metric to assess model performance. The C‐statistic was calculated as “Somers' Dxy rank correlation”/2+0.5. and the 95% CI for the C‐statistic were calculated using the survival (https://github.com/therneau/survival) and CsChange (https://github.com/cran/CsChange) packages in R 4.3.3 (R Foundation for Statistical Computing, Vienna, Austria), using the bootstrap method (with 1000 replicates). When comparing prediction of models with additional parameters, for example, the addition of PPG indices to classical risk factors, the change in C‐statistic, 95% CI for the change in C‐statistic, and *P* value for change in C‐statistic were calculated using the CsChange package with the *P* value calculated by the Z testing method as previously described.^24^ The training and testing/developing data ratio for all machine learning analyses was 8:2. Except where stated, analysis was performed using STATA 18 (StataCorp, College Station, TX) statistical software.

## RESULTS

**Figure 2:**
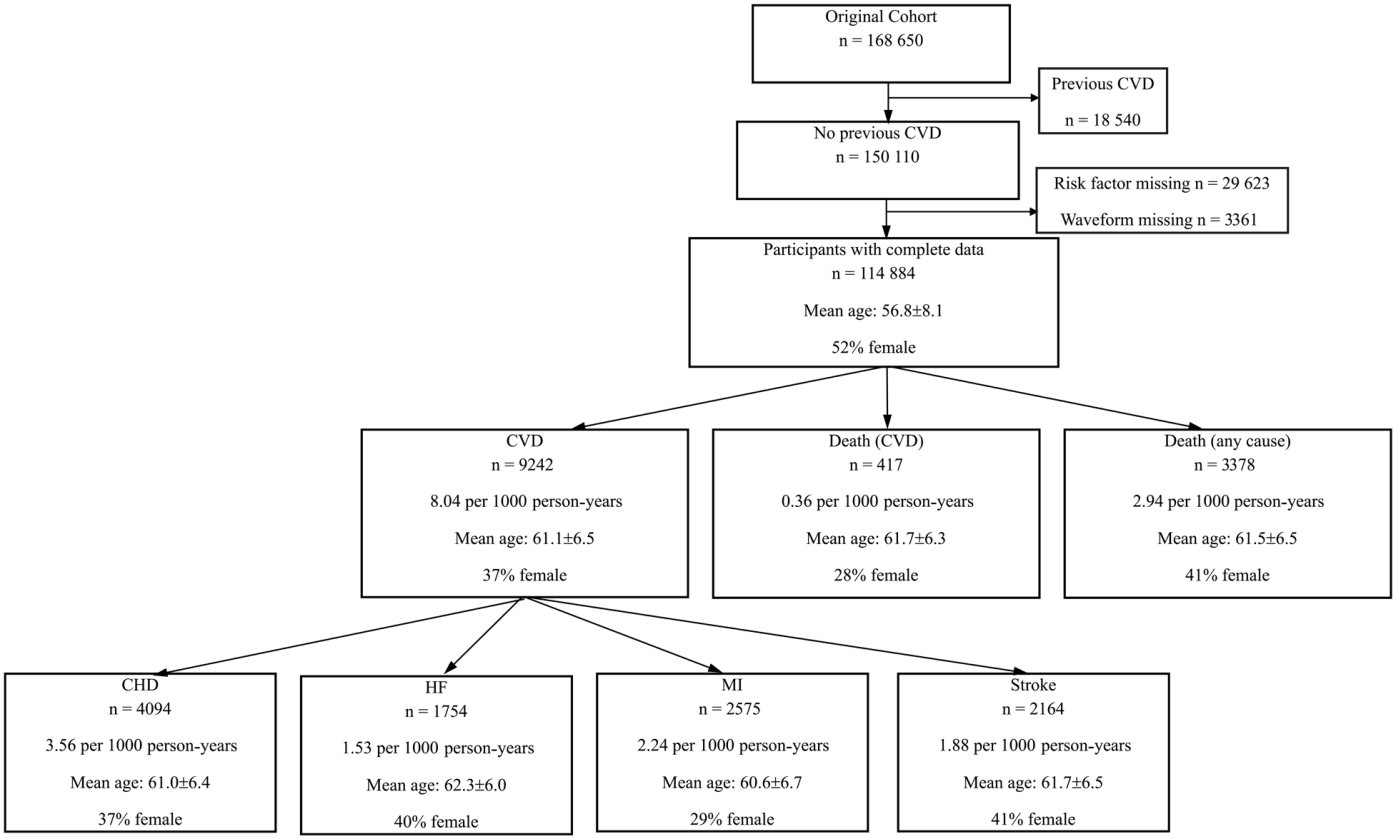
Incident events in the UK Biobank cohort. CHD indicates coronary heart disease; CVD, cardiovascular disease; HF, heart failure; and MI, myocardial infarction.

A total of 168 650 participants whose pulse waveforms were recorded were identified. Participants who had experienced a cardiovascular event as defined previously before their first assessment were excluded (18 540 participants), leaving 150 110 participants, as shown in Figure 2. After removing participants with missing data on waveform indices (3361 participants) or risk factors (29 623 participants missing total‐cholesterol to HDL‐cholesterol ratio or antihypertension medication information), 114 884 participants remained (Figure 2). The mean age of the participants was 56.8±6.1 years, 52% of them were women. Of these, 9242 developed CVD events of which 4094 were a diagnosis of coronary heart disease, 2164 stroke, 1754 heart failure, and 2575 myocardial infarction. During the follow‐up period 3378 participants died from any cause, with CVD recorded as the primary cause of death in 417.

### Relationship of PPG Indices to CVD Events and Mortality

**Figure 3:**
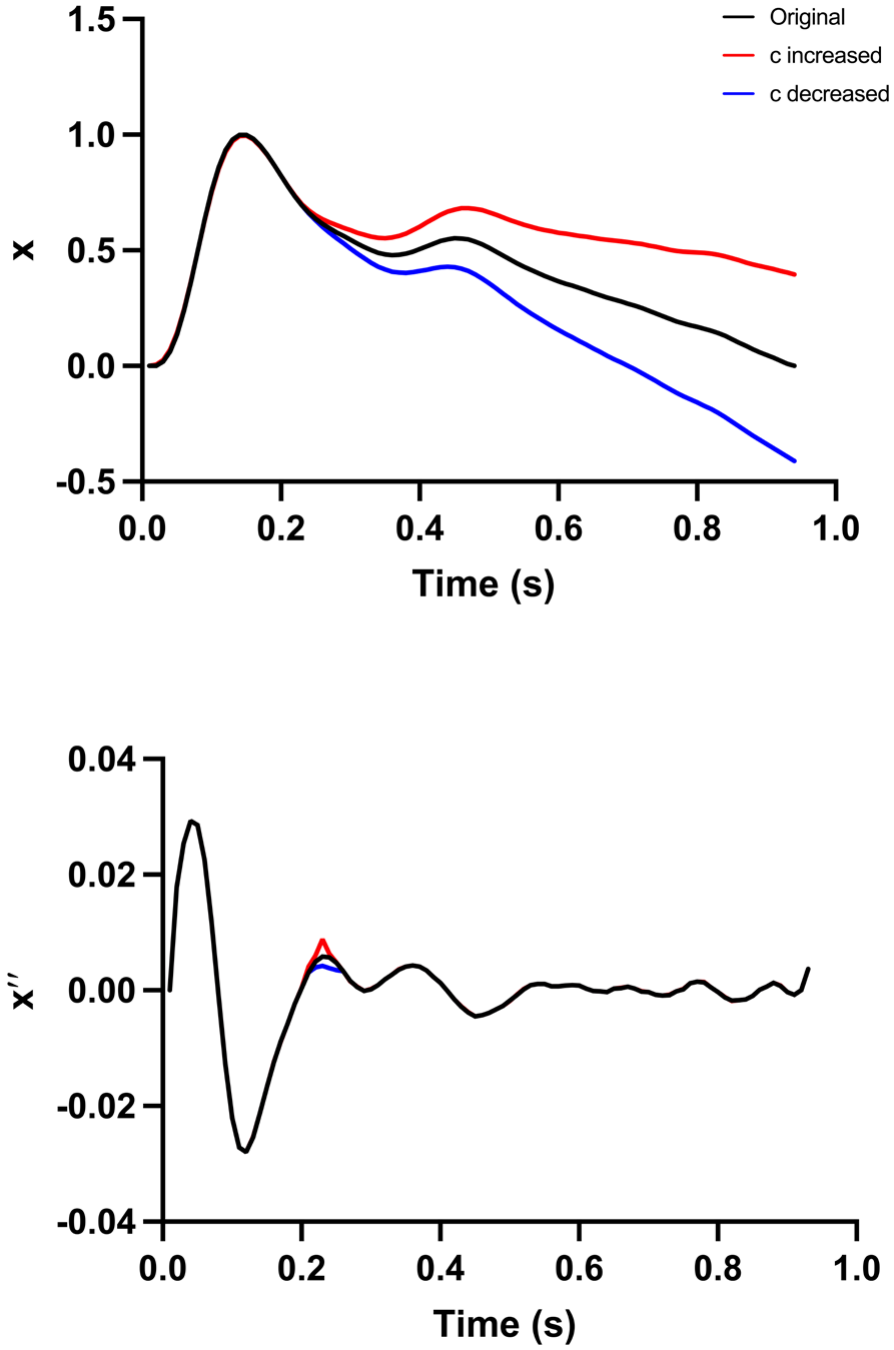
Simulation demonstrating how an increase (red) or decrease (blue) in the c index of the second derivative of the PPG signal (lower) is associated with a change in curvature or deceleration of the mid‐late systolic portion of the PPG (upper). PPG indicates photoplethysmographic pulse waveform.

After adjusting for risk factors (ethnicity, age, sex, BMI, smoking status, presence of diabetes, cholesterol/HDL ratio, mean SBP, and presence of antihypertensive medication), a large number of PPG indices were significantly associated with CVD events and mortality when tested individually (Table 2). These included timing measures, time of systolic peak, time between points b and c of the second derivative of the PPG, and the time between points b and d of the second derivative of PPG signal; amplitudes, peak of first derivative of the PPG, and the peaks a, b, c, d of the second derivative; and more complex indices such as the modified aging index, the slope of a straight line between peaks b and c of the second derivative of the PPG and the slope of a straight line between peaks b and d of the second derivative, and the stiffness constant derived from the PPG (for which *P* for CVD events, not adjusted for multiple testing, <0.001 Table 2). However, the strength of the associations was relatively weak with the majority of the standardized HRs being between 0.9 and 1.1. The strongest association was for the c peak of the second derivative, a measure of curvature of the systolic portion of the waveform (Figure 3), which was significantly negatively associated with CVD events, cardiac and all‐cause mortality with HR of 0.88 (95% CI, 0.85–0.90), 0.74 (95% CI, 0.64–0.85), and 0.83 (95% CI, 0.79–0.87) respectively (all *P*<0.001).

**Table 2:** Association of PPG Indices With Cardiovascular Events, Cardiac and All‐Cause Mortality

PPG index/SD	CVD events			Cardiac death			All‐cause mortality		
	HR	95% CI	*P* value	HR	95% CI	*P* value	HR	95% CI	*P* value
Duration of cardiac cycle (T)	1.02	1.00–1.05	0.027	0.87	0.79–0.96	0.007	0.81	0.78–0.84	<0.001
Time between diastolic and systolic peaks (DT)	0.97	0.94–1.00	0.008	1.04	0.90–1.20	0.593	1.05	1.00–1.10	0.063
Time of systolic peak (CT)	1.05	1.02–1.07	<0.001	0.94	0.84–1.05	0.261	0.90	0.86–0.93	<0.001
Duration of systole (t_systole_)	1.03	1.00–1.06	0.051	0.89	0.77–1.03	0.110	0.86	0.81–0.90	<0.001
Time between points b and c on second derivative (x") of PPG signal (t_b‐c_)	0.95	0.93–0.98	<0.001	0.92	0.81–1.04	0.168	0.91	0.87–0.95	<0.001
Time between points b and d on x″ (t_b‐d_)	0.95	0.93–0.97	<0.001	0.84	0.74–0.95	0.007	0.87	0.83–0.91	<0.001
Amplitude of diastolic peak (dia_amp_)	1.00	0.97–1.03	0.889	0.84	0.74–0.95	0.005	0.84	0.80–0.88	<0.001
Systolic area from pulse foot to dicrotic notch (A1)	1.00	0.97–1.03	0.990	0.84	0.73–0.97	0.014	0.83	0.79–0.88	<0.001
Diastolic area from dicrotic notch to pulse end (A2)	1.01	0.99–1.04	0.393	0.86	0.76–0.96	0.009	0.80	0.77–0.83	<0.001
Amplitude of peak of first derivative (x') of PPG signal (ms)	0.93	0.91–0.95	<0.001	0.97	0.87–1.09	0.622	1.07	1.03–1.11	<0.001
Amplitude of point a on x″ (a)	0.95	0.93–0.97	<0.001	1.01	0.90–1.12	0.919	1.13	1.09–1.17	<0.001
Amplitude of point b on x″ (b)	1.10	1.07–1.13	<0.001	1.07	0.94–1.21	0.351	0.95	0.91–0.99	0.011
Amplitude of point c on x″ (c)	0.88	0.85–0.90	<0.001	0.74	0.64–0.85	<0.001	0.83	0.79–0.87	<0.001
Amplitude of point d on x″ (d)	0.93	0.91–0.96	<0.001	0.88	0.78–0.99	0.032	0.86	0.83–0.90	<0.001
Amplitude of point e on x″ (e)	0.99	0.97–1.02	0.510	1.04	0.93–1.17	0.490	1.10	1.06–1.15	<0.001
Modified aging index (AGI_mod_)	1.16	1.13–1.20	<0.001	1.23	1.06–1.42	0.005	1.10	1.05–1.16	<0.001
d/dt of straight line between points b and c on x″, normalized by amplitude of point a (slope_b‐c_)	0.88	0.85–0.91	<0.001	0.79	0.67–0.94	0.008	0.93	0.88–0.98	0.008
d/dt of straight line between points b and d on x″, normalized by amplitude of point a (slope_b‐d_)	0.90	0.88–0.93	<0.001	0.92	0.81–1.05	0.211	0.99	0.95–1.03	0.632
Ratio of diastolic to systolic area plus ratio of amplitudes of points d to a (IPAD)	0.96	0.94–0.99	0.005	0.85	0.75–0.96	0.011	0.80	0.77–0.84	<0.001
Stiffness constant (k)	0.93	0.91–0.95	<0.001	1.00	0.90–1.11	0.950	1.06	1.03–1.10	<0.001

Adjusted for ethnicity, age, sex, body mass index, smoking status, presence of diabetes, cholesterol/high‐density lipoprotein ratio, mean systolic blood pressure, and presence of antihypertensive medication. CVD indicates cardiovascular disease; HR, hazard ratio per SD change in PPG feature; and PPG, photoplethysmographic pulse waveform.

**Figure 4:**
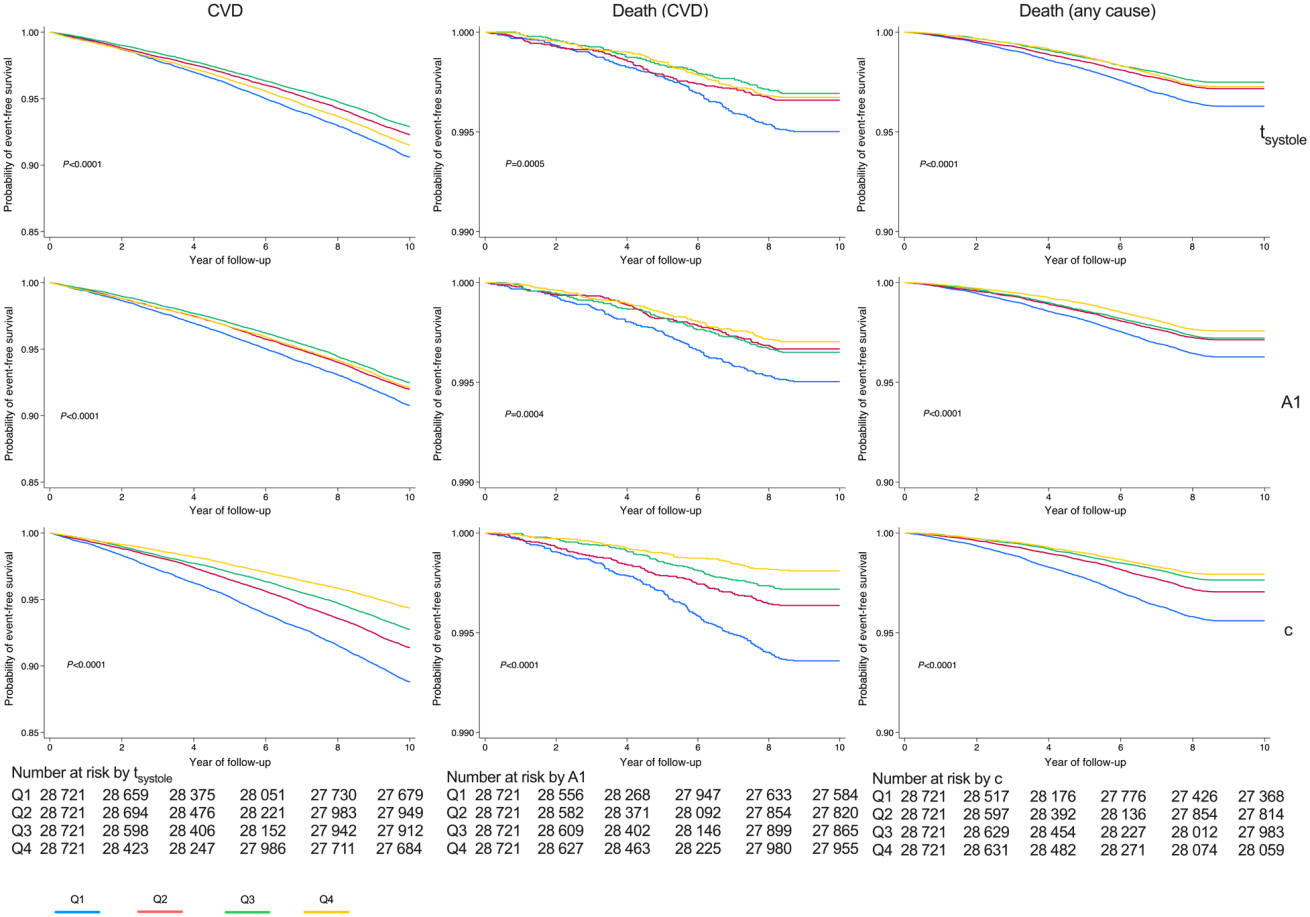
Kaplan–Meier survival curves for PPG indices systolic time, area under the systolic portion of the waveform, and curvature of the midsystolic portion of the waveform. CVD indicates cardiovascular disease; and PPG, photoplethysmographic pulse waveform.

When using the penalized Cox model to examine all PPG indices associated with events, the c peak of the second derivative remained significantly negatively associated with CVD events (HR, 0.80 [95% CI, 0.74–0.86], *P*<0.001). Systolic time was positively associated with CVD events (HR, 1.43 [95% CI, 1.28–1.61], *P*<0.0001) and area under the systolic portion of the waveform (A1) negatively associated with CVD events (HR, 0.79 [95% CI, 0.72–0.88], *P*<0.0001), and the other PPG indices were less strongly associated with CVD events (Table 3). The c peak of the second derivative was the only PPG index significantly associated with cardiac death (HR, 0.72 [95% CI, 0.59–0.89], *P*<0.0001). Kaplan–Meier plots showing the association of the PPG indices systolic time, A1, and c with CVD events are shown in Figure 4. The modified aging index was strongly associated with all‐cause mortality and waveform period and diastolic amplitude were less strongly associated with all‐cause mortality.

**Table 3:** Association of PPG Features With Cardiovascular Events, Cardiac Mortality, and All‐Cause Mortality: Features Selected by Multivariable Elastic Net Penalized Cox Regression

PPG Index/SD	HR	95% CI	*P* value
CVD events			
Duration of cardiac cycle (T)	1.07	1.03–1.12	0.001
Time of systolic peak (CT)	0.90	0.86–0.95	<0.001
Duration of systole (t_systole_)	1.43	1.28–1.61	<0.001
Time between points b and c on x" (t_b‐c_)	1.01	0.97–1.06	0.542
Time between points b and d on x" (t_b‐d_)	1.06	1.00–1.12	0.068
Systolic area from pulse foot to dicrotic notch (A1)	0.79	0.72–0.88	<0.001
Amplitude of point c on x" (c)	0.80	0.74–0.86	<0.001
Amplitude of point d on x″ (d)	0.86	0.82–0.91	<0.001
Amplitude of point e on x" (e)	1.04	0.99–1.10	0.153
Modified aging index (AGI_mod_)	1.09	1.00–1.20	0.062
Ratio of diastolic to systolic area plus ratio of amplitudes of points d to a (IPAD)	1.10	1.04–1.16	0.001
CVD death			
Time between points b and d on x" (t_b‐d_)	1.03	0.87–1.21	0.750
Amplitude of point c on x" (c)	0.72	0.59–0.89	0.002
All‐cause death			
Duration of cardiac cycle (T)	0.90	0.85–0.96	0.001
Time of systolic peak (CT)	0.90	0.81–1.00	0.059
Time between diastolic and systolic peaks (DT)	0.98	0.87–1.10	0.714
Time between points b and d on x" (t_b‐d_)	1.07	1.00–1.15	0.068
Amplitude of diastolic peak (dia_amp_)	0.85	0.77–0.93	0.001
Amplitude of point c on x" (c)	0.94	0.86–1.04	0.218
Amplitude of point e on x" (e)	1.05	0.98–1.12	0.164
Modified aging index (AGI_mod_)	1.31	1.17–1.46	<0.001

Adjusted for ethnicity, age, sex, body mass index, smoking status, presence of diabetes, cholesterol/high‐density lipoprotein ratio, mean systolic blood pressure, and presence of antihypertensive medication. HR, hazard ratio per SD change in PPG feature; PPG, photoplethysmographic pulse waveform.

When assessing the prediction of events, the addition of PPG indices to classical risk factors modestly increased the C‐statistic (Table 4) but did so to a comparable or greater degree than many conventional risk factors such as smoking status, presence of diabetes, lipid profile, and SBP. With age alone the C‐statistic for CVD events was 0.666 and increased by 0.044, 0.002, 0.013, 0.006, 0.004, 0.001, 0.009, and 0.001 to 0.725 after addition of classical risk factors sex, ethnicity, BMI, smoking, diabetes, total‐cholesterol/HDL‐cholesterol, antihypertensive treatment, and SBP respectively. With the addition of PPG features the C‐statistic increased by 0.005 to 0.730. PPG indices contributed more to prediction of events in younger compared with older people (Table 5) for those <50 years, the addition of PPG indices to classical risk factors increased the C‐statistic for CVD events by 0.020 from 0.734 to 0.753 whereas in those ≥65 years it increased the C index by only 0.007 from 0.634 to 0.640 (Table 5). For cardiovascular death and all‐cause mortality, the effect was greater with the addition of PPG indices to classical risk factors increasing the C‐statistic by 0.048 and 0.047 for cardiovascular death and all‐cause mortality in people <50.

**Table 4:** C‐Statistic for Survival Prediction Models Incorporating Classical Risk Factors and PPG Waveform Features

Model	Variables in model	CVD events			CVD death			All‐cause death		
		C‐statistic (95% CI)	ΔC (95% CI) ×10^−2^	*P* value for ΔC	C‐statistic (95% CI)	ΔC (95% CI) ×10^−2^	*P* value for ΔC	C‐statistic (95% CI)	ΔC (95% CI) ×10^−2^	*P* value for ΔC
1	Age	0.666 (0.660–0.671)			0.685 (0.661–0.708)			0.677 (0.668–0.685)		
2	Model 1 + sex	0.689 (0.684–0.694)	4.42 (3.72–5.03)	<0.001	0.727 (0.705–0.749)	4.23 (2.60–5.77)	<0.001	0.687 (0.679–0.696)	1.04 (0.72–1.33)	<0.001
3	Model 2 + ethnicity	0.691 (0.686–0.696)	0.19 (0.09–0.26)	<0.001	0.728 (0.705–0.750)	0.069 (0.22–0.15)	0.474	0.689 (0.680–0.697)	0.15 (0.04–0.30)	0.002
4	Model 3 + body mass index	0.705 (0.700–0.710)	1.34 (1.13–1.56)	<0.001	0.744 (0.722–0.766)	1.60 (0.64–2.63)	0.002	0.691 (0.683–0.700)	0.24 (0.08–0.40)	0.003
5	Model 4 + smoking	0.711 (0.706–0.716)	0.60 (0.45–0.74)	<0.001	0.774 (0.754–0.794)	3.02 (1.61–4.35)	<0.001	0.711 (0.703–0.719)	1.98 (1.54–2.41)	<0.001
6	Model 5 + diabetes	0.714 (0.709–0.719)	0.36 (0.25–0.46)	<0.001	0.787 (0.767–0.806)	1.27 (0.51–2.08)	0.002	0.714 (0.706–0.722)	0.30 (0.14–0.46)	<0.001
7	Model 6 + total cholesterol/high‐density lipoprotein ratio	0.715 (0.710–0.720)	0.11 (0.05–0.17)	<0.001	0.787 (0.768–0.807)	0.09 (−0.14–0.29)	0.409	0.714 (0.706–0.722)	0.008 (−0.022–0.032)	0.581
8	Model 7 + BP medication	0.724 (0.719–0.729)	0.86 (0.70–1.02)	<0.001	0.791 (0.772–0.810)	0.36 (−0.06–0.76)	0.085	0.715 (0.707–0.724)	0.11 (0.01–0.20)	0.023
9	Model 8 + mean systolic BP	0.725 (0.720–0.730)	0.13 (0.07–0.19)	<0.001	0.797 (0.778–0.815)	0.56 (0.02–1.04)	0.032	0.715 (0.707–0.724)	0.021 (−0.029–0.062)	0.367
10	Model 9 + PPG indices	0.730 (0.725–0.735)	0.49 (0.32–0.58)	<0.001	0.809 (0.791–0.827)	1.27 (0.17–1.74)	0.002	0.726 (0.718–0.734)	1.08 (0.70–1.32)	<0.001

BP indicates blood pressure; CVD, cardiovascular disease; PPG, photoplethysmographic pulse waveform; and ΔC, change in C‐statistic compared with previous model.

**Table 5:** C‐Statistic for Survival Prediction Models Incorporating Classical Risk Factors and PPG Waveform Features in Different Age Groups

	CVD events			CVD death			All‐cause death		
	C‐statistic (95% CI)	ΔC (95% CI) ×10^−2^	*P* value for ΔC	C‐statistic (95% CI)	ΔC (95% CI) ×10^−2^	*P* value for ΔC	C‐statistic (95% CI)	ΔC (95% CI) ×10^−2^	*P* value for ΔC
Age <50 y (n=25 852)									
CRF	0.734 (0.715–0.752)			0.833 (0.765–0.901)			0.667 (0.630–0.704)		
CRF+PPG indices	0.753 (0.736–0.771)	1.97 (0.76–2.57)	<0.001	0.880 (0.828–0.932)	4.80 (0.02–8.51)	0.027	0.715 (0.681–0.748)	4.70 (1.57–6.51)	<0.001
50 ≤ age < 65 y (n=66 268)									
CRF	0.690 (0.683–0.697)			0.782 (0.754–0.809)			0.666 (0.653–0.679)		
CRF+PPG indices	0.696 (0.690–0.703)	0.61 (0.33–0.77)	<0.001	0.803 (0.777–0.828)	2.10 (0.03–3.05)	0.007	0.683 (0.671–0.696)	1.73 (0.87–2.15)	<0.001
Age ≥ 65 y (n=22 764)									
CRF	0.634 (0.625–0.643)			0.709 (0.673–0.745)			0.626 (0.611–0.640)		
CRF+PPG indices	0.640 (0.631–0.649)	0.66 (0.18–0.85)	<0.001	0.726 (0.690–0.762)	1.68 (−1.49–2.36)	0.088	0.635 (0.621–0.650)	0.98 (0.001–1.31)	0.006

CRF indicates classical risk factors including age, sex, body mass index, diabetes, smoking status, ethnic background, cholesterol/high‐density lipoprotein ratio, mean SBP, and BP medication; CVD, cardiovascular disease; and PPG, photoplethysmographic pulse waveform.

## DISCUSSION

The present study confirms previous work^8^, ^10^ that the PPG contains prognostic information. The association with CVD events, cardiovascular death, and all‐cause mortality remained after adjustment for classical risk factors including SBP and confirms prognostic prediction additional to that mediated through that associated with classical risk factors. The major novel finding is the identification of individual features most strongly associated with incident CVD events. These relate to the systolic portion of the PPG. Less time in systole as estimated from fiducial points in the PPG and, for a given time in systole, greater area under the PPG during systole were both associated with fewer CVD events. Greater curvature of the PPG in mid‐late systole was associated with both CVD events and CVD deaths. Features such time between the first and second peak (used with subject height to derive stiffness index), previously related to arterial stiffness^23^ did not contribute to prediction of events. The features identified in the present study likely relate to efficient contraction of the left ventricle, with a greater proportion of ventricular ejection occurring in early systole (left ventricular [LV] first‐phase ejection fraction), leading to a shortening of systole and greater early systolic deceleration.^25^ LV first‐phase ejection fraction appears a sensitive measure of LV dysfunction and has powerful prognostic impact in conditions associated with increased ventricular load.^26^ Within the UK Biobank population, the condition leading to most events is likely to be atherosclerosis and ischemic heart disease. It is notable that the presence of ischemic heart disease is associated with impaired LV first‐phase ejection fraction^27^ and it may be that this, rather than arterial stiffness, is what mediates the association between PPG features and CVD events. Other previous work on the PPG has focused on measures of cardiovascular aging obtained by relating PPG features to chronological age,^11^ assuming such features are then more closely related to biological rather than chronological age. It is notable that the PPG aging index derived from several features of the photoplethysmography was strongly related to all‐cause mortality. It is possible that the characteristics of cardiovascular aging captured by the PPG aging index are indicative of generalized biological aging. The present study provides proof‐of‐concept that features of the PPG relating to systolic function and cardiovascular aging are of prognostic importance. Further work involving the comparison of the PPG with imaging data and measures of biological age will be required to confirm the mechanisms underlying the association between PPG features and cardiovascular events and all‐cause mortality. This will provide information on which conditions, for example early subclinical heart failure, the PPG is most efficient at detecting and the link between cardiovascular and biological aging.

When considering the utility of PPG features in risk prediction, the addition of PPG indices to classical risk factors resulted in only a small improvement in the accuracy of the prediction of CVD events. However, this small improvement in risk prediction is similar to that from a recent study in which deep learning was applied to the PPG waveform^10^ and suggests that the features we have identified contribute to the majority of the prognostic information that can be extracted from the PPG. Importantly, although the improvement in the predictive accuracy for CVD events was small, it was comparable to or greater than that produced through the addition of many classical risk factors such as presence of diabetes, lipid profile, and systolic BP. The clinical utility in prediction of events was more marked in younger compared with older people indicating that the PPG might be useful in providing screening information in middle‐aged people. In the context of a measurement that can be derived with no cost or inconvenience (eg, the estimated 30% of the population who wear “smart watches”), the ability to detect even a small proportion of the population that may be at increased risk is potentially important. Furthermore, there is considerable potential to improve on the potential for risk stratification using “wearable” devices. The PPG is subject to variability due to recording artifact and physiological state of arousal. The same limitations apply to a measurement of “office” BP from a mean of 2 to 3 oscillometric measurements and it is notable that PPG features improved risk classification more than SBP when the PPG was recorded for only 10 to 15 seconds. The ability to record the PPG continuously over indefinite periods of time via wearable technology has potential to reduce measurement noise and physiological variability with consequent improvement in risk prediction. Such improvement in risk prediction is likely to exceed that provided by ambulatory over office BP because the time over which prognostic information will be extracted from the PPG will increase by many orders of magnitude. However, we cannot exclude the possibility that adjustment of PPG features for more accurate measurement of representative BP by ambulatory BP would reduce the association of PPG features with CVD events.

Strengths of the present study are the large cohort size and relatively long follow‐up period giving a large number of CVD events. The main limitation, apart from those discussed of short recording duration, is the lack of a validation cohort. We are not aware of another larger cohort with PPG and outcome data and validation in specific target groups will be required. This limitation is somewhat mitigated by the large cohort size and that effects in the same direction are seen in different age strata of the cohort. Finally, it should be noted that prediction cannot test a causal association of PPG features with clinical outcomes.

### Conclusions

In conclusion, the PPG signal that can be derived from wearable digital health technology has the potential to detect people at high risk of CVD events, probably because of LV systolic dysfunction.

## Sources of Funding

This work was supported by a research grant from the Medical Research Council (MR/W026198/1).

## Disclosures

None.

## Supporting information

Table S1
