# Supplementary material for: Prediction of Cardiovascular Disease Events From the Photoplethysmograph Waveform
Source: J Am Heart Assoc. 2025 Dec 3;14(24):e040237. doi: 10.1161/JAHA.124.040237 (PMC12826887; doi:10.1161/JAHA.124.040237)
Supplement: Supplementary file 1 — Table S1 [file JAH3-14-e040237-s001.pdf]

Supplementary Materials

Table S1. Indices obtained from the PPG

Feature	Description in words	Formula
**PPG signal x: amplitudes**		
dia_{amp}	Diastolic peak amplitude	x(dia)
**x: timings**		
T	Duration of cardiac cycle	
$t_{systole}$	Duration of systole	t(dic)
CT	Time of systolic peak	t(s)
DT	Time between diastolic and systolic peaks	t(dia)-t(s)
**x: areas and ratios**		
A1	Systolic area from pulse foot to dicrotic notch	
A2	Diastolic area from dicrotic notch to pulse end	
**PPG first derivative x’: amplitudes and ratios**		
ms	Maximum slope, amplitude of first derivative	x’(ms)
**PPG second derivative x”: ratios of amplitudes**		
a	Amplitude of 'a'	x’’(a)
b	Amplitude of 'b'	x’’(b)
c	Amplitude of 'c'	x’’(c)
d	Amplitude of 'd'	x’’(d)
e	Amplitude of 'e'	x’’(e)
AGI_{mod}	Ageing index: modified	(x”(b)-x”(c)-x”(d))/x”(a)
**x”: timings**		
t_{b-c}	Time between b and c	t(c)-t(b)
t_{b-d}	Time between b and d	t(d)-t(b)
**x”: slopes**		
$slope_{b-c}$	*d*/*dt* of straight line between *b* and *c*, normalised by *a*	
$slope_{b-d}$	*d*/*dt* of straight line between *b* and *d*, normalised by *a*	
**Multiple indices**		
IPAD	Ratio of diastolic to systolic area plus amplitude of ‘d’ relative to ‘a’	(A2/A1)+ x”(d)/x”(a)
k	Stiffness constant	x”(s)/((x(s)-x(ms))/x(s))
**Indices not used**		
t_{dia}	Duration of diastole	T-t(dic)
$prop_s$	Ratio of time of systolic peak over cardiac period	t(s)/T
t_{ratio}	Ratio of systolic to diastolic durations	t(dic)/(T-t(dic))
$prop_{\Delta t}$	Ratio of ΔT over cardiac period	(t(dia)-t(s))/T
IPA	*Ratio of diastolic area over systolic area*	A2/A1
b/a	Amplitude of ‘b’ relative to ‘a’	x”(b)/x”(a)
c/a	Amplitude of ‘c’ relative to ‘a’	x”(c)/x”(a)
d/a	Amplitude of ‘d’ relative to ‘a’	x”(d)/x”(a)
e/a	Amplitude of ‘e’ relative to ‘a’	x”(e)/x”(a)

Definitions: t, time since pulse onset; x, PPG signal; x’, PPG first derivative; x”, PPG second derivative.
